# p53-Dependent Transcriptional Responses to Interleukin-3 Signaling

**DOI:** 10.1371/journal.pone.0031428

**Published:** 2012-02-14

**Authors:** Anissa M. Jabbour, Lavinia Gordon, Carmel P. Daunt, Benjamin D. Green, Chung H. Kok, Richard D'Andrea, Paul G. Ekert

**Affiliations:** 1 Children's Cancer Centre, Murdoch Children's Research Institute, Royal Children's Hospital, Parkville, Victoria, Australia; 2 Cell Signalling and Cell Death, Walter and Eliza Hall Institute of Medical Research, Parkville, Victoria, Australia; 3 Department of Paediatrics, University of Melbourne, Royal Children's Hospital, Parkville, Victoria, Australia; 4 Bioinformatics Unit, Murdoch Children's Research Institute, Royal Children's Hospital, Parkville, Victoria, Australia; 5 Acute Leukaemia Laboratory and Department of Haematology, SA Pathology, Department of Haematology and Oncology, The Queen Elizabeth Hospital, Centre for Stem Cell Research, the University of Adelaide, Adelaide, South Australia, Australia; German Cancer Research Center, Germany

## Abstract

p53 is critical in the normal response to a variety of cellular stresses including DNA damage and loss of p53 function is a common feature of many cancers. In hematological malignancies, *p53* deletion is less common than in solid malignancies but is associated with poor prognosis and resistance to chemotherapy. Compared to their wild-type (WT) counterparts, hematopoietic progenitor cells lacking *p53* have a greater propensity to survive cytokine loss, in part, due to the failure to transcribe Puma, a proapoptotic Bcl-2 family member. Using expression arrays, we have further characterized the differences that distinguish *p53^−/−^* cells from WT myeloid cells in the presence of Interleukin-3 (IL-3) to determine if such differences contribute to the increased clonogenicity and survival responses observed in *p53^−/−^* cells. We show that *p53^−/−^* cells have a deregulated intracellular signaling environment and display a more rapid and sustained response to IL-3. This was accompanied by an increase in active ERK1/2 and a dependence on an intact MAP kinase signaling pathway. Contrastingly, we find that *p53^−/−^* cells are independent on AKT for their survival. Thus, loss of *p53* in myeloid cells results in an altered transcriptional and kinase signaling environment that favors enhanced cytokine signaling.

## Introduction

p53 is a critical regulator of the response to DNA damage and oncogenic stress. Loss of p53 function, through mutation or deletion, is a frequent occurrence in human malignancies. In hematological malignancies, p53 deletion, 17p-, is less common, but is a poor prognostic feature. p53 functions to regulate several pathways, including cell cycle arrest, DNA repair and apoptosis through transcriptional upregulation of proapoptotic Bcl-2 genes, in particular Puma/Bbc3 and Noxa [1], [2], [3], [4], [5], [6], [7]. Loss of p53 protects cells from p53-dependent apoptotic stimuli due to limited Puma and Noxa transcriptional upregulation.

The induction of apoptosis is a key tumor suppressor function of p53, particularly in those cells which acquire other oncogenic lesions [8]. p53-dependent Puma upregulation has a central role in this response, inducing apoptosis in the transformed cells [9]. Interestingly, in response to an acute DNA-damaging stress such as ionizing radiation, p53-dependent upregulation of Puma may actually contribute to tumor development in some models [10], [11]. In this situation, p53-dependent apoptosis induces cell death in thymic cells which have sustained DNA damage but not yet acquired oncogenic mutations. This cell loss creates a niche into which surviving cells with transforming mutations may proliferate.

It is increasingly apparent that p53 also has a critical role in regulating the response to a wide variety of cellular stresses. For example, we and others have shown that deletion of *p53* can protect cells against apoptosis induced by cytokine deprivation, in particular Interleukin-3 (IL-3) deprivation [12], [13]. These results complement earlier observations from Lotem and Sachs [14], who showed that untransformed hematopoietic progenitor cells from *p53^−/−^* mice formed colonies in limiting doses of cytokine. *p53*-deficient hematopoietic progenitor cells were also able to form more colonies in the absence of IL-3 compared to wild-type (WT) cells [12]. These experiments clearly suggest that p53 is involved in modulating the sensitivity to cytokine with a loss of p53 increasing viability and growth in the presence of limiting cytokine doses. The p53 response is also coupled to cell death in the absence of cytokine with a failure to upregulate Puma expression a likely explanation for this [12]. Thus, in the presence of IL-3 receptor signaling, a p53-dependent response modulates the sensitivity to cytokine receptor activation and may also contribute to growth and survival differences important for tumorigenesis.

To explore this further we compared the expression profiles of WT cells and *p53^−/−^* IL-3 dependent cells [12], hereafter referred to as FDM (Factor Dependent Myeloid) cells, in the presence or absence of IL-3, using microarray analysis. Under normal culture conditions, *p53* deleted cells have substantially different gene expression profiles compared to WT cells. Some of these differences are in genes that regulate cytokine signaling, in particular genes such as *SOCS1* and *SOCS3*, and the cell cycle inhibitor *p21*. The different gene expression profiles suggest that even under optimal growth conditions deletion of *p53* alters gene expression rendering cells more responsive to changes in cytokine levels. This may in part explain our and others observation that lower doses of IL-3 are required to maintain viability of *p53^−/−^* cells compared to WT cells [14]. In support of this hypothesis, we show that MAP Kinase signaling is activated earlier and in a more sustained manner in *p53^−/−^* cells after IL-3 stimulation. Interestingly, we also observed that *p53^−/−^* cells treated with an AKT inhibitor were protected from cell death in comparison to WT cells indicating that AKT activation is redundant. In comparison, *p53^−/−^* cells were sensitive to an MEK inhibitor indicating that MAP Kinase signaling was required for *p53^−/−^* viability.

Expression array analysis of IL-3 withdrawal responsive genes by Signaling Pathway Impact Analysis (SPIA) of curated pathways indicates that WT samples displayed an identifiable response with pathways such as the JAK-STAT, Insulin and p53 signaling pathways significantly altered. In contrast, the changes in gene expression in *p53^−/−^* cells upon IL-3 withdrawal did not show the alterations to downstream cytokine signaling. Thus, the down-modulation of cytokine signaling on withdrawal of cytokine appears to be p53-dependent.

## Materials and Methods

### Generation of IL-3 dependent FDM cells

Murine WT and *p53^−/−^* factor dependent myeloid (FDM) cells were generated previously by HoxB8 transformation [12] and their generation was approved by the animal ethics committee at the Murdoch Children's Research Institute (AEC 594) and Walter & Eliza Hall Institute (2003.024). All FDM cells were cultured in DMEM (low glucose; Gibco) supplemented with 10% fetal calf serum (FCS; JRH Laboratories) plus 0.25 ng/mL IL-3 (R&D systems).

### Expression array

Expression array was performed as previously described [12]. Briefly, RNA from three biological replicates per genotype was isolated using Qiagen RNAeasy extraction kit according to the manufacturer's instruction. RNA was labeled, amplified and hybridized to Illumina MouseWG-6 V1 Expression BeadChips according to Illumina standard protocols. Samples were processed at the Australian Genome Research Facility, Melbourne, Australia. A total of 18 arrays comprising over 46,000 transcripts that interrogates the whole mouse genome, were generated covering three time points (0, 6, 18 hours after IL-3 deprivation), and each time series repeated in triplicate with independent biological samples (Gene Expression Omnibus: NCBI gene expression and hybridization array data repository accession GSE18770).

### Expression array analysis

The data were analyzed using the lumi package [15] from Bioconductor [16] and R (R development core, http://www.R-project.org).

### Expression array quality assessment

The quality of the array series was assessed using the package *arrayQualityMetrics*, which offers an extensive set of visualizations and metrics for assessing microarray data quality [17]. Applied to this dataset, *arrayQualityMetrics* indicates that although in general the data are of good quality, one sample was considered to be an outlier and hence was excluded from further analysis *(arrayQualityMetrics)*. The proportion of expressed probes for each array is represented in Table 1.

**Table 1:** Proportion of expressed probes for each array.

Genotype and hours withdrawn of IL-3	WT 0 h	WT 6 h	WT 18 h	*p53^−/−^* 0 h	*p53^−/−^* 6 h	*p53^−/−^* 18 h
Proportion of expressed probes	0.319	0.340	0.330	0.319	0.319	0.301

The empirical reliability of each array following a linear model fit was estimated. A normexp-by-control background correction, quantile normalization and log2 transformation was carried out. Probes that failed to achieve a BeadStudio detection p value of 0.01 on any array were deemed to be not expressed, and hence were removed from all subsequent analyses. Hierarchical clustering was carried out which is hierarchical cluster analysis on a set of dissimilarities. These dissimilarities are a distance matrix computed by using the Manhattan distance on the expression data. A linear model was fitted for each probe; subsequently differential expression analysis was carried out.

### Signaling Pathway Impact Analysis (SPIA)

For the signaling pathways available in KEGG (Kyoto Encyclopedia of Genes and Genomes), the Signaling Pathway Impact Analysis (SPIA) algorithm [18], [19], [20] was conducted. SPIA uses information from differentially expressed genes and their fold changes, as well as pathways topology in order to assess the significance of the pathways in the condition under the study. At time of writing, 80 pathways were available for searching. A false discovery adjusted global p-value cut-off of 0.1 was chosen. A SPIA analysis provides information on the KEGG pathway being perturbed (the KEGG ID), the number of genes in the KEGG pathway (pSize), the number of differentially expressed genes found within the pathway (NDE), the False Discovery Rate (pGFdr), and whether the pathway is activated or inhibited (Status).

### Gene Set Enrichment Analysis (GSEA)

Broad GSEA software was used to analyze array results [21], [22]. A mean-rank gene set test to analyze whether a set of genes is highly ranked relative to other genes in terms of the residual standard deviation for each gene taken from the linear model fitted to the array. Genes were considered expressed in the array based on their detection p-value.

### Western blot analysis

Cells were lysed in RIPA buffer (150 mM NaCl, 50 mM TrisHCl pH 7.4, 0.5% sodium deoxycholate (DOC), 0.1% SDS, 1% NP40, protease inhibitor cocktail, 5 mM ßglycerophosphate, 1 mM Na Molybdate, 2 mM Na pyrophosphate, 10 mM NaF). Lysates were resolved by SDS-PAGE and immunoblotted with the following antibodies: rabbit monoclonal anti-phospho Stat5 (Tyr 694; Cell Signaling), rabbit monoclonal anti-Stat5 (Cell Signaling), rabbit polyclonal anti-phospho ERK1/2 (Thr 202/Tyr 204; Cell Signaling), rabbit polyclonal anti-ERK1/2 (Cell Signaling), rabbit monoclonal anti-phospho AKT (Ser 473; Cell Signaling), mouse monoclonal anti-AKT (Cell Signaling), mouse monoclonal anti-ßactin, goat polyclonal anti-mouse IgG coupled to HRP (Sigma Aldrich) and donkey polyclonal anti-rabbit IgG coupled to HRP (Amersham).

### Cell Viability Assays

IL-3 was removed by washing cells 3 times in PBS before being cultured in DMEM/10% FCS with or without IL-3 (as indicated) or treated with the AKT inhibitor VIII (Calbiochem) at 0, 0.1 or 0.5 ng/mL or a MEK inhibitor (U0126; Selleck chemicals) at 0, 40 or 200 nM. Cell viability was determined by staining cells with Propidium Iodide (PI, 1 µg/mL; Sigma- Aldrich) followed by flow-cytometric analysis (LSR II; Becton Dickinson).

### Clonogenic survival assays

Clonogenic survival assays were performed as previously described [23], [24]. Briefly, 1×10^4^ cells/mL were cultured in DMEM, 10% FCS with or without IL-3 at various concentrations. After three days, cells were plated in 6-well plates in DMEM, 20% FCS, 0.5 ng/mL IL-3, and 0.3% soft agar. After 14 days the numbers of colonies were counted and expressed as a percentage relative to the number of colonies generated per 1000 cells cultured in IL-3. At least 3 independent clones of each genotype were assayed in each experiment.

## Results

### Gene expression profile of WT and *p53^−/−^* FDM cells in the presence of IL-3

**Figure 1:**
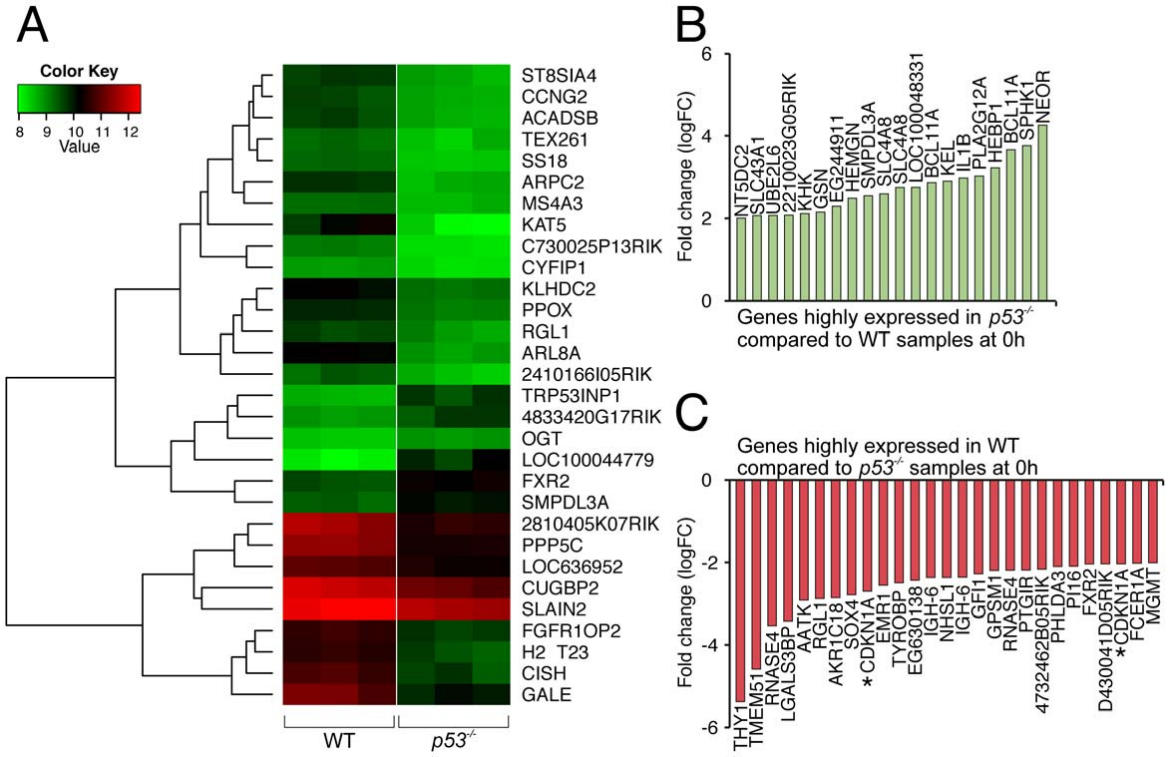
Differential gene expression in WT and *p53^−/−^* FDM samples. (A) RNA from three independent WT and *p53^−/−^* FDM cell lines were analyzed with the use of the 6-chip Illumina expression array. The heatmap depicts the expression of the top 30 differentially expressed genes according to the adjusted P value in WT and *p53^−/−^* samples. (B) Differentially expressed genes highly expressed in *p53^−/−^* compared to WT samples with a logFC change of greater than 2 are shown. (C) Differentially expressed genes highly expressed in WT compared to *p53^−/−^* samples with a logFC change of greater than 2 are shown. Asterisks show the p53-dependent gene CDKN1A (p21).

Previously, we showed that the apoptotic response to cytokine deprivation in immortalized IL-3 dependent myeloid cells (Factor Dependent Myeloid or FDM cells) differed significantly between cells derived from WT and *p53^−/−^* mice. Decreased Puma protein levels in *p53^−/−^* cells contributed to the survival advantage exhibited by these cells when starved of IL-3 [12]. We speculated that p53-dependent transcription under non-stress conditions (abundant IL-3) also contributed to the enhanced viability and clonogenicity of *p53^−/−^* FDM cells. As previously described [12], RNA samples from three independent clones each of WT and *p53^−/−^* FDM cells, 0, 6 and 18 h after IL-3 withdrawal, were analyzed using the Illumina MouseWG-6 Expression BeadChip array. To extract the most significantly variant genes, we considered that significantly differentially expressed genes had an adjusted p-value of 0.1 or lower and a positive B statistic (1000 probes). In the presence of IL-3, 533 probes, representing 519 genes, showed significantly higher expression in the WT samples compared to *p53^−/−^* samples and 467 probes representing 431 genes showed significantly reduced expression. The top 30 most differentially expressed genes in WT and *p53^−/−^* samples are shown in Figure 1A. Three members of the Suppressor of Cytokine Signaling (SOCS) family, the SH2-containing protein, CISH (also known as CIS or SOCS), SOCS1 and SOCS3 were expressed at significantly higher levels in WT cells, compared to *p53^−/−^* cells in the presence of IL-3. The SOCS family of proteins are modulated by various cytokines including IL-2, IL-3, EPO and G-CSF [25]. CISH functions to inhibit STAT5, in the presence of IL-3, resulting in reduced activation of the JAK/STAT pathway [26]. This raises the possibility that in the absence of p53 decreased CISH expression results in amplified IL-3 signaling. To determine whether mRNA levels of SOCS1 and SOCS3 reflected difference in protein expression, we probed lysates from 4 independent WT and 4 independent *p53^−/−^* cell lines with antibodies to SOCS1 and SOCS3 (supplemental Figure S1A). These data show that SOCS3 expression varies between cell lines but does not show any consistent elevation in *p53^−/−^* cells. Although SOCS1 expression was in fact higher in 2 of 4 *p53^−/−^* cell lines than in WT cell lines, these differences were not consistent across all cell lines. In addition, we saw no consistent differences in expression of IL-3 receptor components between WT and *p53^−/−^* cell lines. These data suggest that differences in the response to IL-3 signaling between WT and *p53^−/−^* cell lines is not explained by expression levels of the IL-3 receptor, SOCS1 or SOCS3.

To highlight the most prominent p53-dependent genes under these conditions we selected genes with a LogFc of greater than 2, since this would identify genes whose expression was much higher in *p53^−/−^* than WT (potentially repressed by p53), and genes whose expression was much higher in WT than *p53^−/−^* (potentially p53-induced) (Figure 1B and 1C). As expected, well-described p53-dependent genes such as p21 (Cdkn1a) were expressed at significantly lower levels in *p53^−/−^* FDM samples compared to WT, indicating our system correctly identified known p53-dependent genes. The genes most significantly over-expressed by fold-change in *p53^−/−^* cells other than the Neomycin resistance cassette, were Sphk1, Sphingosine Kinase 1 and Bcl11A suggesting that these are normally down-regulated by p53. Down-regulation of Sphk1 by p53 may be important for the apoptotic response as Sphk1 expression is associated with increased survival [27] and has been described in association with activated PI3K/AKT signaling [28], [29]. Bcl11A is a zinc-finger transcription factor, which is an essential regulator of lymphopoiesis in mice, and its elevated expression has been associated with some lymphoma patients in humans [30], [31], [32], [33]. Interestingly, Chronic Lymphocytic Leukemia samples with gains at 2p16 locus (mapping to REL and Bcl11a) are more frequent in samples which also bear 17p- (p53 loss) deletions [34], [35]. The mechanism of this correlation is unknown. In our FDM cells, although we identified an increase in Bcl11a mRNA in *p53* null samples, protein levels of Bcl11a were not consistently elevated in multiple *p53^−/−^* FDM clones compared to WT FDM clones cultured in the presence of IL-3 (Supplementary Figure S1B). Only subtle elevations of isoform 1 (84 kda) and isoform 5 (53 kda) were observed in *p53^−/−^* FDM clones.

### Pathway Analysis

**Figure 2:**
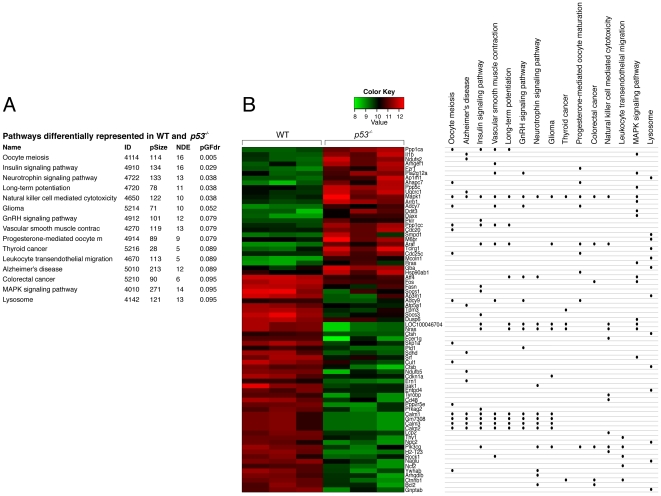
Pathway analysis of WT and *p53^−/−^* samples cultured in the presence of cytokine. (A) List of activated and inactivated pathways identified by a Signaling Pathway Impact Analysis (SPIA) of array results from WT and *p53^−/−^* samples. The ID is the KEGG ID, pSize indicates the number of genes in the KEGG pathway, NDE is the number of differentially expressed genes found within the pathway and pGFdr is the False Discovery pathways (FDR<0.1). Significant pathways are shown. (B) The differentially expressed genes that account for the significant SPIA pathways (from Figure 2A) are depicted by the heatmap. To the right of the heatmap, the dots indicate the pathways to which each gene contributes and whether a gene is represented by several of the pathways.

A Signaling Pathway Impact Analysis (SPIA) algorithm [18], [19], [20] was performed for the pathways available in KEGG (Kyoto Encyclopedia of Genes and Genomes). SPIA uses information from a set of differentially expressed genes and their fold changes, as well as pathways topology, to assess the potential significance of a specific pathway being altered in a gene list comparison. A false discovery adjusted global p-value cut-off of 0.1 was chosen. The fifteen pathways that were differentially represented in *p53^−/−^* and WT FDM cells are listed in Figure 2A. Since the same genes may be represented in many different pathways, we were interested in which differentially expressed genes accounted for the various pathway calls (Figure 2B). A relatively small group of differentially expressed genes accounted for the many activated or inactivated pathways. For example, all isoforms of Calmodulin (Calm1, Calm2 and Calm3) were represented in 7 different pathways, and were decreased in *p53^−/−^* cells. In total, there were 73 differentially expressed genes within these fifteen pathways, with 46 upregulated in WT and 27 upregulated in *p53* null samples. MAPK1 expression is represented in all but 2 of the 15 pathways, and was upregulated in *p53^−/−^* cells. The Insulin signaling pathway and MAPK signaling pathways were identified as pathways which are selectively activated in *p53^−/−^* FDM cells suggesting these are normally down-modulated by p53 (Figure 2B). The genes included in these pathways are also known to be involved in cytokine signal transduction such as IL-3 signaling and includes PIK3 gamma (PIK3CG), SOCS1 and 3, MAPK1 and a-Raf. Activation of these pathways in *p53^−/−^* FDM cells is consistent with a de-regulated signal transduction environment and raises the possibility that *p53^−/−^* cells are intrinsically more responsive to IL-3 and other cytokines.

### p53-dependent gene family enrichment in FDM samples

**Figure 3:**
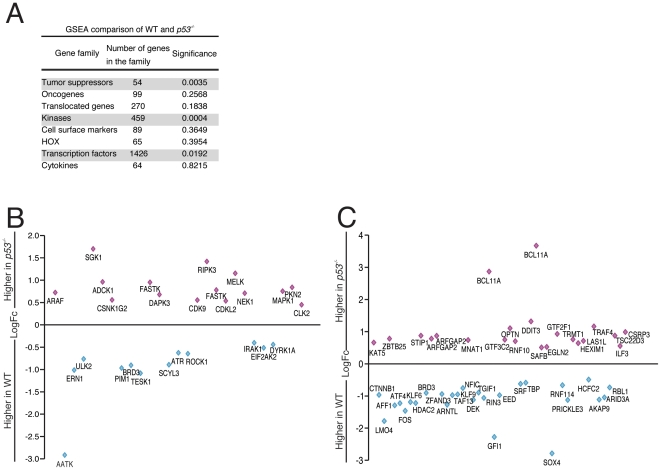
Gene set enrichment analysis of WT and *p53^−/−^* expression array data. (A) Gene Set Enrichment Analysis (GSEA) of the WT and *p53^−/−^* gene lists at the various time points. GSEA was conducted using all genes that were considered expressed in the array, based on their detection p-value. A p-value of less than 0.05 was considered significant. (B) (C) Each diamond represents an individual probe for significantly differentially expressed kinases (B) or transcription factors (C) in WT and *p53^−/−^* FDM cells cultured in IL-3.

Gene Set Enrichment Analysis (GSEA) was done to compare families of genes whose expression most differed between WT and *p53^−/−^* FDM cells. The analysis was performed on the listed gene families in Figure 3A [36], [37], [38], [39], [40], [41]. The results indicated that the gene families most different between WT to *p53^−/−^* FDM samples were the tumor suppressors, kinases and transcription factors. The logFC changes of each of individual differentially expressed kinases are shown in Figure 3B. SGK1 (serum/glucocorticoid kinase 1), and RIPK3 were expressed at approximately a 1.5 fold higher level in *p53^−/−^* cells, suggesting p53 functions to regulate the expression of these kinases under physiological conditions. However, RIPK3 protein levels in p53 null lysates were higher than seen in WT cells in 2 of 4 cell lines (Supplemental Figure S1A). SGK1 is a serine-threonine kinase related to AKT/PKB involved in transducing cytokine-dependent survival signals [42] and like Sphk1 its down-regulation may be important for an apoptotic response. In contrast, RIPK3 is a kinase involved in necroptosis activated by TNF Receptor Family signaling.

As mentioned above, Bcl11a was significantly upregulated in *p53^−/−^* samples. In WT samples, the lineage-specific transcription factor GFI1 was expressed at approximately 2-fold greater levels relative to *p53^−/−^* cells suggesting that p53 levels may contribute lineage choices. GFI1 may repress hematopoietic colony formation and Bcl11A over-expression is associated with proliferative signals and some AML subtypes [43], [44]. Together, these data suggest that a combination of an activated intracellular signaling environment, combined with the expression of transcription factors which favor clonogenic proliferation, contribute to the survival and proliferative advantages observed in *p53^−/−^* cells.

### FDM transcriptional response to cytokine withdrawal

We also extended the analysis of changes in gene expression in WT and *p53^−/−^* FDM cells after IL-3 deprivation beyond the analysis of Bcl-2 family members we have previously published [12]. We used a linear model to determine which genes were differentially expressed in WT or *p53^−/−^* at 0 h compared to 6 h and 6 h compared to 18 h post cytokine withdrawal (with the resulting t-statistics classifying genes as either up, down or not significant).

**Figure 4:**
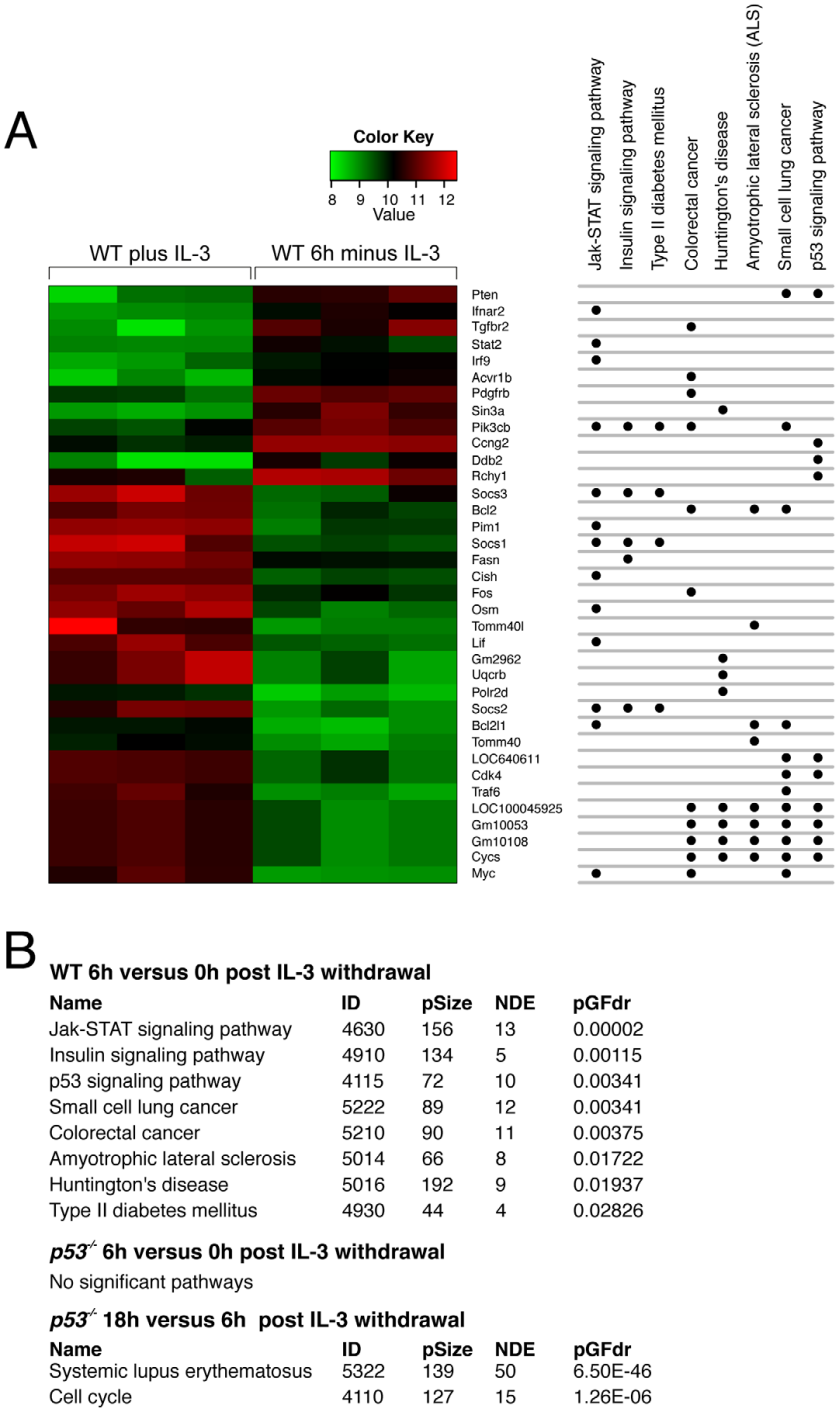
Differential pathway expression in WT samples after IL-3 loss. (A) Three independent WT and *p53^−/−^* FDM cell lines were culture with or without IL-3 for 6 h. RNA was extracted and expression array was performed as in Figure 1A. The heat map shows significant changes in expression after IL-3 deprivation in WT cells and dots indicate the various SPIA pathways these represent. 31 differentially expressed genes were active in these pathways, with 24 being highly expressed at time zero and 12 highly expressed at 6 h IL-3 withdrawal. Significant pathways are shown (FDR<0.1). (B) Comparison of SPIA of array results from WT and *p53^−/−^* samples after IL-3 withdrawal. The ID is the KEGG ID, pSize indicates the number of genes in the KEGG pathway, NDE is the number of differentially expressed genes found within the pathway and pGFdr is the False Discovery pathways (FDR<0.1). Significant pathways are shown.

The pathways most affected by 6 h of IL-3 withdrawal were identified by SPIA KEGG analysis (Figure 4A and B). As anticipated, the JAK/STAT, Insulin and p53 signaling pathways were the top 3 pathways identified by SPIA to be responding to IL-3 withdrawal. This type of analysis is not a definitive indication of signaling kinase pathway activation, since the activity of such pathways is dictated by events other than just gene transcription. However, it is interesting that p53-dependent pathways were identified as inactivated in this analysis, indicating p53-dependent transcription contributes to the normal response to IL-3 signaling, as well as the apoptotic response to IL-3 deprivation.

In contrast, the transcriptional differences observed *p53^−/−^* FDM samples assayed after 6 h of IL-3 deprivation did not cluster in the same SPIA pathways as WT cells (Figure 4B). By 18 h, only two KEGG pathways in the *p53* null cell lines were activated (Figure 4 and Supplementary Figure S2).

### Increased sensitivity of *p53^−/−^* FDM cells to IL-3

**Figure 5:**
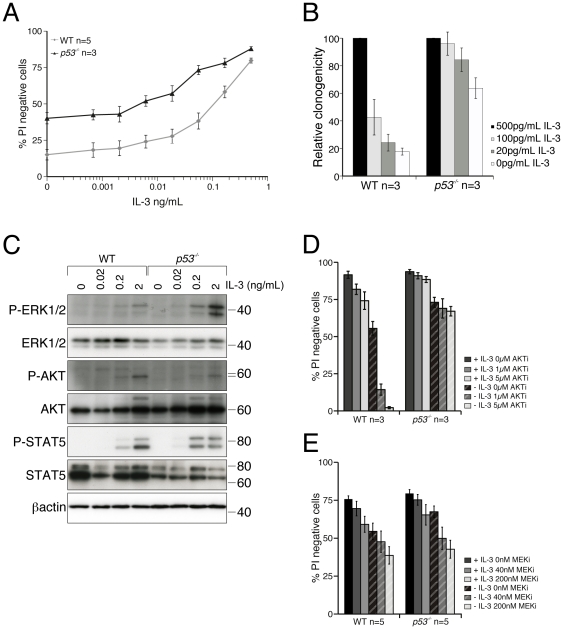
Analysis of the IL-3 signaling pathway in WT and *p53^−/−^* FDM cells. (A) Independently generated WT and *p53^−/−^* FDM cell lines (n = number of cell lines) were cultured in the indicated concentrations of IL-3 for 72 hours. Viability was determined using Propidium iodide (PI) exclusion detected by flow cytometry. Results show the mean +/− SEM of 3 independent experiments. (B) WT and *p53^−/−^* independent FDM cell lines (n = number of cell lines) were cultured in the indicated concentrations of IL-3 doses for 72 hours and then plated in soft agar with abundant IL-3. The number of colonies was counted after 14 days and the clonogenicity (relative to the number of colonies generated in 500 pg/ml IL-3) calculated. Results show the mean +/− SEM of 2 independent experiments. (C) Lysates were generated from WT pr *p53^−/−^* FDM cells following stimulation with the indicated concentrations of IL-3 after 16 hours of IL-3 deprivation. Lysates were resolved by SDS-PAGE and immunoblotted with antibodies specific to the indicated proteins. (D) Independent WT and *p53^−/−^* FDM cell lines (n = number of cell lines) were treated for 24 h with an AKT inhibitor (AKTi) in the presence or absence of IL-3. Viability was determined by flow cytometric analysis of PI exclusion. Results show the mean +/− SEM of 2 independent experiments. (E) Independent WT and *p53^−/−^* FDM cell lines (n = number of cell lines) were treated for 24 h with a MEK inhibitor (MEKi) in the presence or absence of IL-3. Viability was determined by flow cytometric analysis of PI exclusion. Results show the mean +/− SEM of 4 independent experiments.

We wanted to determine if the altered pathway activation or inhibition revealed by gene expression profiling translated into detectable differences in cellular responses to IL-3 signaling. One prediction was that *p53^−/−^* cells would initiate IL-3 receptor signaling at lower concentrations of IL-3 than WT cells. To test this, we compared the viability of WT and *p53^−/−^* FDM cells in decreasing concentrations of IL-3 ligand in both viability and clonogenic assays (Figure 5A and 5B). At all except the highest concentrations of IL-3 used, more *p53^−/−^* FDM cells were viable than WT cells (Figure 5A). After WT or *p53^−/−^* FDM cells were cultured in limiting IL-3 doses for three days they were plated in soft agar containing abundant IL-3 (Figure 5B). In this clonogenic assay, *p53^−/−^* cells were able to form more colonies than WT cells at limiting IL-3 concentrations. This suggests that IL-3 can transduce survival signals in *p53*-deleted cells at many fold lower concentrations that in WT cells.

To determine if differential activation of cytokine signaling pathways was occurring in *p53^−/−^* FDM cells after IL-3 stimulation, lysates from cells stimulated with various concentrations of IL-3 after a period of IL-3 deprivation were probed with antibodies to detect activation of the signaling kinases AKT, ERK and STAT5 (Figure 5C). The most consistent difference observed was that ERK1/2 phosphorylation occurred in response to lower concentrations of IL-3 in *p53^−/−^* FDM cells (see also Supplementary Figure S3). This was consistent with the array data showing higher ERK2 (MAPK1) mRNA levels of in *p53^−/−^* FDM cells and pathway analysis which suggested activation of the MAPK pathway. There were no differences in total ERK1/2 levels between WT and *p53^−/−^* cells (Figure 5C). This data indicates that loss of *p53* resulted in higher levels of phosphorylated ERK1/2 and ERK1/2 activation occurred in response to lower IL-3 ligand concentrations. As transient activation of the ERK/MAPK pathway can have profound effects on cellular response compared to prolonged activation [45], loss of *p53* may sensitize cells to transient activation of this pathway leading to a substantial increase in cell survival compared to WT cells.

AKT activation, as indicated by phosphorylation of serine 473 after IL-3 stimulation, was comparable between WT and *p53^−/−^* cells, or even slightly blunted in *p53^−/−^* cells (Figure 5C). This suggested the possibility ERK activation was relatively more important in *p53^−/−^* cells than in WT cells. We treated WT and *p53^−/−^* FDM cells, cultured in the presence or absence of IL-3, with increasing concentrations of an AKT or a MEK inhibitor to determine whether *p53^−/−^* and WT cells had altered susceptibility to apoptosis induced by these drugs. In response to the AKT inhibitor, WT cells showed a dose-dependent decrease in viability in the presence of IL-3, which was exacerbated by IL-3 deprivation. Intriguingly, *p53^−/−^* cells were highly resistant to cell death induced by AKT inhibition, in the presence or absence of IL-3. The withdrawal of IL-3 resulted in the death of approximately 15% of *p53^−/−^* FDM cells, but this was not significantly increased by AKT inhibition (Figure 5C). In contrast, the reduction of *p53^−/−^* FDM cell viability was similar to the response of WT cells (Figure 5D). To determine whether AKT inhibition also affected ERK1/2 phosphorylation, or if MEK inhibition affected AKT phosphorylation, we probed western blots of lysates from cells treated with either inhibitor for phosphorylated AKT or ERK1/2 (Supplemental Figure S4). There was no evidence to indicate that AKT inhibitor had an impact on ERK1/2 phosphorylation or the reverse. AKT inhibition caused an increase in Puma expression in WT but not *p53* null cells. Puma is normally upregulated in response to IL-3 deprivation [12]. It is not surprising therefore that inhibition of AKT results in Puma upregulation. This suggests that in this model there no crosstalk between these two pathways. Taken together, these results suggest that IL-3 dependent FDM cells lacking *p53* no longer require AKT signaling to maintain viability, but are, in part at least, dependent on ERK1/2 signaling. This suggests further that regulation of AKT activation in response to IL-3 receptor signaling is p53-dependent.

## Discussion

Although loss of function mutations or deletions affecting p53 is frequent in many tumors, it is relatively rare in *de novo* AML [46] and strongly associated with poorer prognosis. One possible explanation is that p53-dependent proapoptotic proteins such as Puma are important predictors of the responsiveness of many cancer types, including myeloid malignancies, to chemotherapeutic drugs [47]. However, it is also the case that hematopoietic progenitor cells lacking *p53* have a greater capacity for clonal proliferation in the absence of any apoptotic stimuli [12]. Our data demonstrates that deletion of *p53* results in substantial differences in mRNA expression profiles in cells under normal culture conditions, and that the cell biological correlates of these expression differences manifest as enhanced survival in limiting doses of IL-3 and an amplified signaling response to IL-3 stimulation. This altered response to growth factor signaling may contribute to tumorigenesis and altered responses to treatment observed in the absence of p53.

In this study we did not focus on p53-dependent regulation of apoptosis. We, and others, have shown that p53-dependent upregulation of Puma and Noxa is essential for normal apoptosis in response to p53-dependent death stimuli [3], [4], [6], [23], [48]. At the same time, it has been elegantly demonstrated that the failure of cells to undergo apoptosis does not account for all of the oncogenic effects of loss of p53 function [8]. Indeed, in T-cell lymphoma induced by irradiation, p53-dependent apoptosis was required for tumor development, by removing cells and creating a niche in which malignant cells could proliferate [10], [11].

We sought to determine how *p53* null myeloid cells differed from their WT counterparts in the absence of an apoptotic stimulus, and how such differences might contribute to the oncogenic effects of p53 deletion. Expression levels of intracellular kinases such as MAPK and secondary messenger molecules such as the calmodulins suggested that *p53^−/−^* hematopoietic cells have activated signal transduction pathways compared to WT cells. However, expression levels alone do not prove the activation of such pathways, nor whether there is any measurable effect on cell behavior. Strikingly, ERK1/2 was more rapidly phosphorylated at lower IL-3 concentrations in cells without p53. This is an indication that MAPK signaling pathways are more readily activated in *p53^−/−^* cells. It seems probable that a number of factors, other than the expression levels of kinases, contribute to this phenomenon. For example, *p53^−/−^* cells also expressed lower levels of SOCS mRNA and higher levels of RIPK3 which may contribute to cytokine receptor signaling. It is important to note that these mRNA levels did not necessarily correlate with consistent differences in protein expression, emphasizing the importance of validating expression array experiments at a pathway level as well as validating individual proteins. Further, it is also apparent that variations in mRNA expression levels between WT and *p53^−/−^* cells do not necessarily reflect differences in p53 response genes. It is likely that many of the observed differences arise because of adaptation to the lack of p53, triggered by deregulation of a smaller number of direct p53 response genes.

Activation of PI3K and AKT is part of the IL-3 receptor signaling pathways which regulate cell viability in response to IL-3 signaling [49]. Our pathway analyses indicated that PI3K expression levels were lower in *p53^−/−^* FDM cells than WT cells. When we looked for AKT activation in response to IL-3 stimulation, we observed that AKT was phosphorylated in *p53* null cells similarly to WT cells. However, the resistance of *p53^−/−^* FDM cells to apoptosis induced by AKT inhibition suggests that AKT activation is not required for IL-3 survival signaling in *p53^−/−^* cells. AKT activation and p53 regulation are linked by experimental evidence showing the E3 ubiquitin ligase which regulates p53 stability, MDM2, is itself a substrate of AKT [50]. When cytokine receptor activation results in PI3K/AKT activation, MDM2 is phosphorylated and stabilized, resulting in p53 poly-ubiquitination and proteasomal degradation. Our data, and that of others, suggests that the anti-apoptotic functions of AKT, particularly in the context of IL-3 deprivation or deprivation of metabolic substrates such as glucose, depend critically on p53. In the absence of p53, this pathway becomes redundant. Thus, we conclude that p53 plays an important role in the regulation of signal transduction pathways activated by cytokines such as IL-3.

Interestingly, when *p53^−/−^* cells were treated with a MEK inhibitor, they underwent apoptosis similar to WT cells. This indicates that cells lacking *p53* retain a dependence on MAP kinase signaling, which is supported by the increased ERK1/2 phosphorylation in these cells. Proteomic analysis of Acute Myeloid Leukemia samples has indicated that patients with higher levels of p53 (which often is indicative of p53 mutation) also had abundant ERK2 [51]. It is well recognized that loss of *p53* is a poor prognostic feature in hematological malignancy, at least in part because cells lacking functional p53 do not activate apoptosis pathways in response to chemotherapeutic drugs [52]. Our data suggest that an alternative treatment strategy in p53-deleted myeloid tumors would be the use of MAP kinase inhibitors. Our data further suggest targeting of the PI3K/AKT pathway is unlikely to be a successful therapeutic option where p53 is deleted or mutated.

## Supporting Information

Figure S1 **Protein expression of SOCS1, SOCS3, RIPK3, ß common chain, IL-3 α chain and Bcl11a in WT and** ***p53^−/−^*** **FDM cells.** (A and B) Lysates were extracted from multiple clones of WT or *p53^−/−^* FDM cells cultured in IL-3. Lysates were resolved by SDS-PAGE and immunoblotted with antibodies specific the indicated proteins. ßactin is shown as a loading control. (A) Two exposures of ß common are shown to demonstrate expression in WT cell line 4 and *p53^−/−^* cell line 1. (B) Bcl11a isoforms molecular weight are as follows, 1–84 kda, 2–47 kda, 3–27 kda, 4–45 kda, 5–27 kda, 6–21 kda, 7–14 kda, 8–53 kda.
(TIF)

Figure S2 **Differential pathway expression in** ***p53^−/−^*** **samples after IL-3 loss.** *p53^−/−^* FDM cell clones were withdrawn of IL-3 for 6 or 18 h were analyzed by SPIA. Significant pathways are shown (FDR<0.1).
(TIF)

Figure S3 **ERK activation after IL-3 stimulation of WT and** ***p53^−/−^*** **FDM cells.** Lysates were extracted from WT pr *p53^−/−^* FDM cells cultured in the absence of IL-3 for 16 h followed by a 15 minute IL-3 re-addition at various concentrations (as indicated). Lysates were resolved by SDS-PAGE and immunoblotted with antibodies specific to phospho-ERK, total ERK and ßactin.
(TIF)

Figure S4 **AKT inhibition does not alter ERK1/2 phosphorylation and MEK inhibition does not affect AKT phosphorylation.** Lysates were extracted from cells treated with either AKTi or MEKi and resolved on SDS-PAGE and immunoblotted with the indicated antibodies. The predominant isoform of Bim is BimL. An asterisk indicates the correct Puma band. ßactin is shown as a loading control.
(TIF)
