# Supplementary figures and images for: p53-Dependent Transcriptional Responses to Interleukin-3 Signaling
Source: PLoS One. 2012 Feb 14;7(2):e31428. doi: 10.1371/journal.pone.0031428 (PMC3279357; doi:10.1371/journal.pone.0031428)

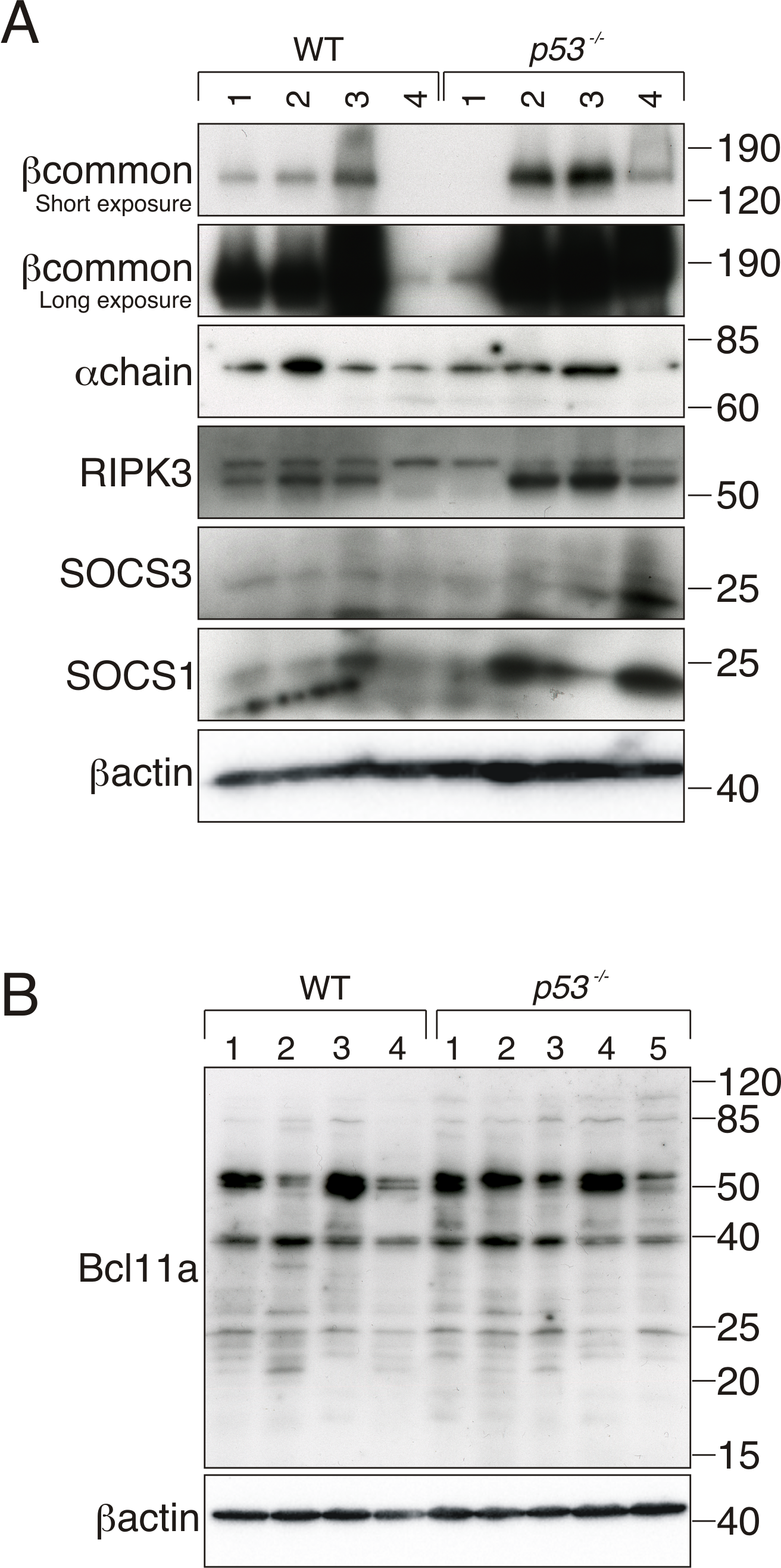

Supplement: Figure S1 — Protein expression of SOCS1, SOCS3, RIPK3, ß common chain, IL-3 α chain and Bcl11a in WT and p53−/− FDM cells. (A and B) Lysates were extracted from multiple clones of WT or p53−/− FDM cells cultured in IL-3. Lysates were resolved by SDS-PAGE and immunoblotted with antibodies specific the indicated proteins. ßactin is shown as a loading control. (A) Two exposures of ß common are shown to demonstrate expression in WT cell line 4 and p53−/− cell line 1. (B) Bcl11a isoforms molecular weight are as follows, 1–84 kda, 2–47 kda, 3–27 kda, 4–45 kda, 5–27 kda, 6–21 kda, 7–14 kda, 8–53 kda. (TIF) [file pone.0031428.s001.tif]

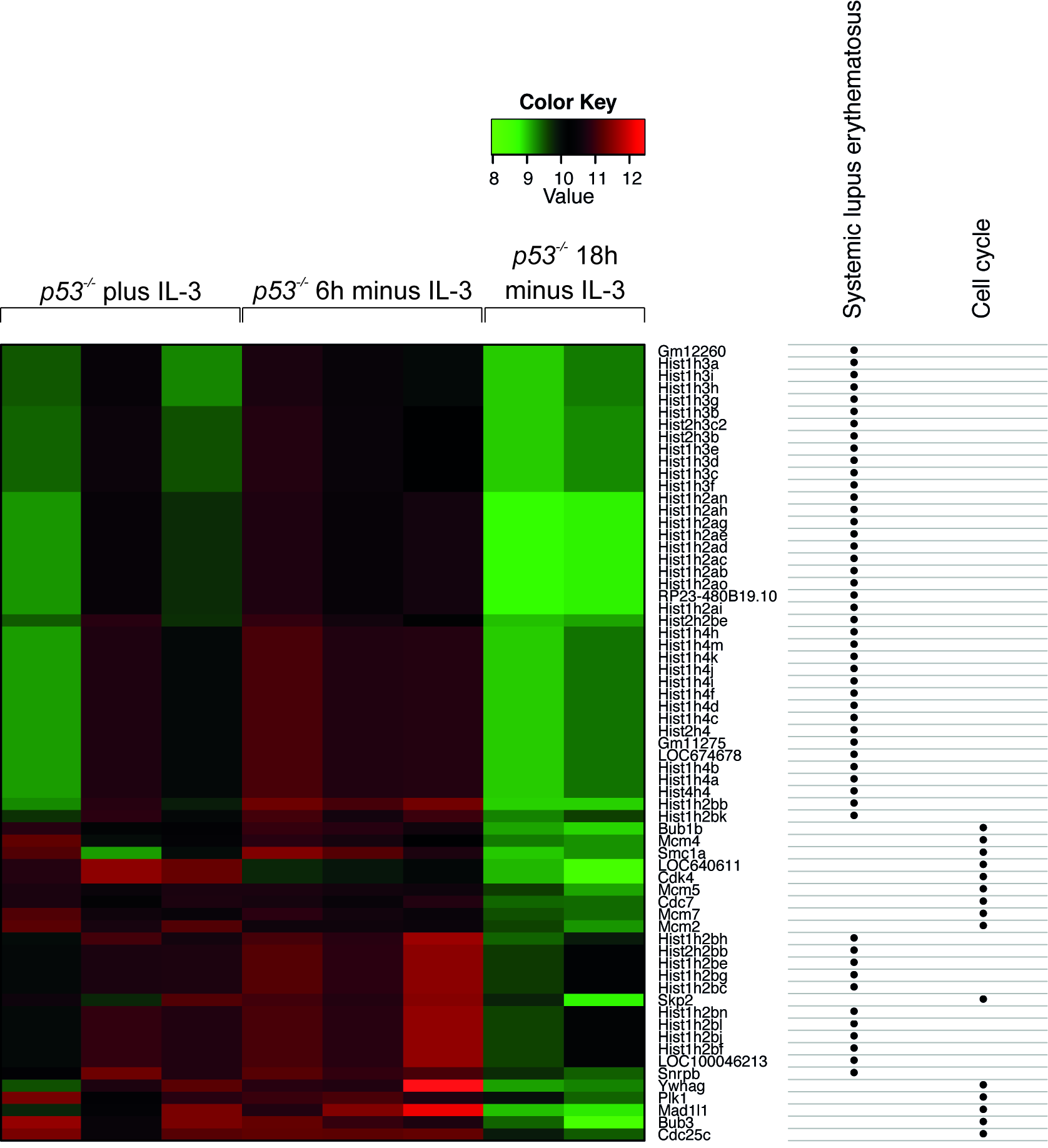

Supplement: Figure S2 — Differential pathway expression in p53−/− samples after IL-3 loss. p53−/− FDM cell clones were withdrawn of IL-3 for 6 or 18 h were analyzed by SPIA. Significant pathways are shown (FDR<0.1). (TIF) [file pone.0031428.s002.tif]

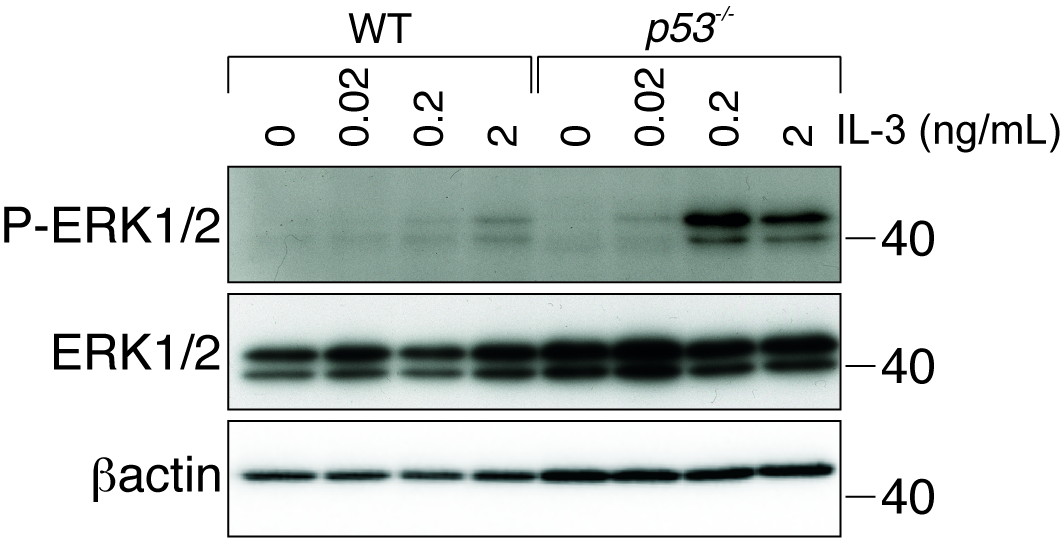

Supplement: Figure S3 — ERK activation after IL-3 stimulation of WT and p53−/− FDM cells. Lysates were extracted from WT pr p53−/− FDM cells cultured in the absence of IL-3 for 16 h followed by a 15 minute IL-3 re-addition at various concentrations (as indicated). Lysates were resolved by SDS-PAGE and immunoblotted with antibodies specific to phospho-ERK, total ERK and ßactin. (TIF) [file pone.0031428.s003.tif]

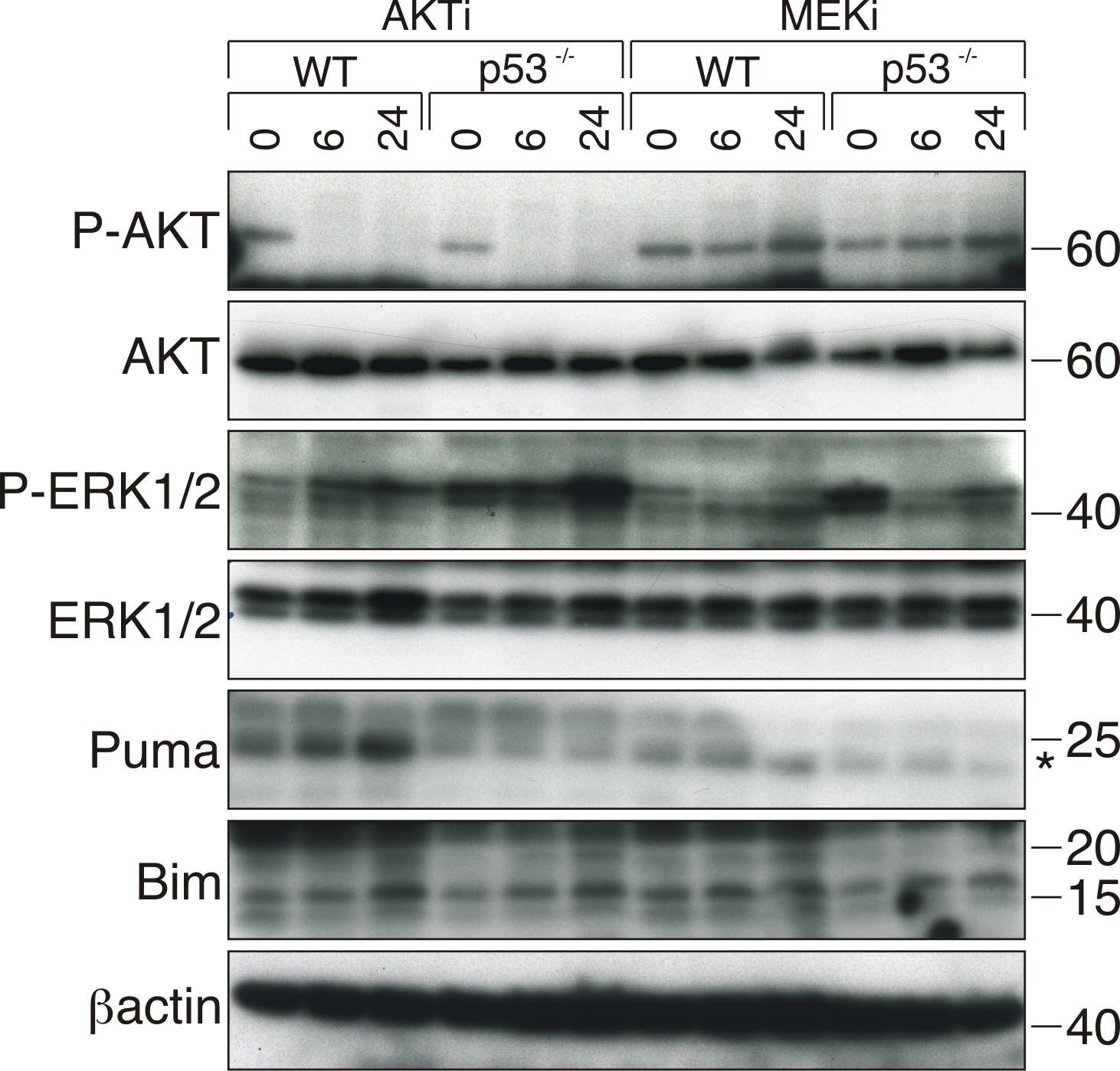

Supplement: Figure S4 — AKT inhibition does not alter ERK1/2 phosphorylation and MEK inhibition does not affect AKT phosphorylation. Lysates were extracted from cells treated with either AKTi or MEKi and resolved on SDS-PAGE and immunoblotted with the indicated antibodies. The predominant isoform of Bim is BimL. An asterisk indicates the correct Puma band. ßactin is shown as a loading control. (TIF) [file pone.0031428.s004.tif]
